# The SOD Mimic MnTnHex-2-PyP^5+^ Reduces the Viability and Migration of 786-O Human Renal Cancer Cells

**DOI:** 10.3390/antiox8100490

**Published:** 2019-10-17

**Authors:** João G. Costa, Nuno Saraiva, Ines Batinic-Haberle, Matilde Castro, Nuno G. Oliveira, Ana S. Fernandes

**Affiliations:** 1 Research Center for Biosciences & Health Technologies (CBIOS), Universidade Lusófona de Humanidades e Tecnologias, Campo Grande 376, 1749-024 Lisboa, Portugal; jgcosta@ulusofona.pt (J.G.C.); nuno.saraiva@ulusofona.pt (N.S.); 2 Research Institute for Medicines (iMed.ULisboa), Faculty of Pharmacy, Universidade de Lisboa, Av. Professor Gama Pinto, 1649-003 Lisboa, Portugal; mcastro@ff.ulisboa.pt (M.C.); ngoliveira@ff.ulisboa.pt (N.G.O.); 3 Department of Radiation Oncology, Duke University School of Medicine, Durham, NC 27710, USA; ibatinic@duke.edu

**Keywords:** SOD mimic, MnTnHex-2-PyP^5+^, reactive oxygen species, cell migration, clear-cell renal carcinoma

## Abstract

Clear-cell renal carcinoma (ccRCC) is the most common type of renal cancer. The importance of oxidative stress in the context of this disease has been described, although there is only little information concerning the role of superoxide dismutase (SOD) enzymes. The importance of SOD in different pathological conditions promoted the development of SOD mimics (SODm). As such, manganese(III) porphyrins can mimic the natural SOD enzymes and scavenge different reactive oxygen species (ROS), thus modulating the cellular redox status. In this study, the exposure of 786-O human renal cancer cells to MnTnHex-2-PyP^5+^ (MnP), a very promising SODm, led to a concentration and time-dependent decrease in cell viability and in the cell proliferation indices, as well as to an increase in apoptosis. No relevant effects in terms of micronuclei formation were observed. Moreover, the exposure to MnP resulted in a concentration-dependent increase in intracellular ROS, presumably due to the generation of H_2_O_2_ by the inherent redox mechanisms of MnP, along with the limited ability of cancer cells to detoxify this species. Although the MnP treatment did not result in a reduction in the collective cell migration, a significant decrease in chemotactic migration was observed. Overall, these results suggest that MnP has a beneficial impact on reducing renal cancer cell viability and migration and warrant further studies regarding SODm-based therapeutic strategies against human renal cancer.

## 1. Introduction

Renal cell carcinoma (RCC) comprises up to 5% of all malignant tumors [1,2,3]. Over the past decade, a substantial amount of new information concerning the epidemiology, molecular and immunologic characteristics of RCC as well as novel therapies has emerged. Several studies suggested a genetic basis for renal cell carcinoma [4,5]. Clear-cell renal carcinoma (ccRCC) is the most common type of renal cancer, accounting for approximately 75% of renal epithelial malignancies [5,6]. The main feature of ccRCC includes a hypoxia status with the activation of angiogenesis. The majority of sporadic ccRCC is associated with defects in von Hippel–Lindau (VHL) tumor suppressor gene [6,7]. Moreover, the activation of the mammalian target of rapamycin (mTOR), a key protein for the cell growth, metabolism and migration has also a crucial role in the pathophysiology of the ccRCC [1,7].

Reactive oxygen species (ROS) have an important role in the initiation, development, and progression of cancer [8,9,10]. Oxidative stress causes direct and irreversible oxidative damage to macromolecules but also disrupts key redox-dependent signaling processes. The presence of high levels of ROS such as hydrogen peroxide (H_2_O_2_), hydroxyl radical (HO**·**), peroxynitrite (ONOO^−^) and superoxide (O_2_^•−^) were already been described in RCC [3]. In ccRCC, oxidative alterations in lipids, proteins, and DNA have also been described [11,12].

It is well-known that cellular antioxidant defenses play a crucial role against oxidative stress. The superoxide dismutase enzymes (SOD) are part of these important natural antioxidant defenses. Many studies show a reduction in SOD expression in various types of cancer, when compared to normal tissues, suggesting a tumor suppressor role for this enzyme. The MnSOD polymorphism (Ala16Ala), which results in a lower activity of SOD, has been associated with an increased susceptibility to develop renal cancer [13]. Conversely, other studies report an elevation in MnSOD expression in cancer, supporting a role for this enzyme in the progression of tumors to a more aggressive stage [11,14,15,16]. These differences are possibly related to the differential cellular levels of H_2_O_2_ and MnSOD. 

The increasing understanding of the role of SOD in physiological and pathological conditions resulted in the development of synthetic compounds with the capacity to mimic the native enzyme [17]. SOD mimics (SODm) are able to catalytically remove O_2_^•−^ through a dismutation process and scavenge different types of reactive species. The mode of action of SODm was initially considered to be highly specific towards O_2_^•−^. Nevertheless, in the last years, with an increase in the knowledge of the cellular oxidative stress processes, SOD mimics have been pointed out as significant redox modulators in different redox-sensitive signaling pathways [18]. Therefore, SODm have the ability to affect proliferation, differentiation and cell death [18]. Manganese(III) porphyrins (MnPs) are a particular group of SODm that have exhibited beneficial effects in different pathological conditions related to oxidative stress. Moreover, some of these compounds also display an important therapeutic potential in cancer therapy as radio- and chemosensitizers, as well as radioprotectors of normal tissue [19]. MnTnHex-2-PyP^5+^ is considered one of the most promising SODm. Besides the lipophilicity and biocompatibility, it has also excellent bioavailability. In addition, the pharmacokinetic studies revealed appropriate tissue penetration and retention with a preference for mitochondria. Moreover, MnTnHex-2-PyP^5+^ has a large therapeutic window as demonstrated by in vivo studies [18].

Despite the promising results of MnPs in different types of cancer, there are no available studies addressing the role of this particular class of redox drugs in the context of renal cancer. Therefore, this work intends to fill this gap, being the first attempt to study the potentially valuable effect of MnTnHex-2-PyP^5+^ toward the treatment of renal cancer, integrating multiple endpoints of cell viability, apoptosis, genotoxicity, induction of ROS and cell migration.

## 2. Materials and Methods

### 2.1. Chemicals

RPMI-1640 medium was obtained from ATCC (Manassas, VA, USA). Fetal bovine serum (FBS), phosphate buffered saline (PBS, 0.01 M, pH 7.4), trypsin, penicillin-streptomycin (pen/strep) solution, crystal violet, dimethylsulfoxide (DMSO), cytochalasin-B and RNAse were obtained from Sigma-Aldrich (St. Louis, MO, USA). The TrypLE™ Express Enzyme solution was obtained from Gibco, Invitrogen (UK). The (3-(4,5-dimethylthiazol-2-yl)-5-(3-carboxymethoxyphenyl)-2-(4-sulphophenyl)-2*H*-Tetrazolium)-CellTiter 96^®^ AQueous One Solution Reagent (MTS) was obtained from Promega Corp. (Madison, WI, USA). The Giemsa dye, methanol, ethanol, acetic acid and propidium iodide (PI) were purchased from Merck (Darmstadt, Germany). The dihydrorhodamine 123 (DHR) and the Alexa Fluor^®^ 488 Annexin V/PI kit were acquired from Molecular Probes (Eugene, OR, USA). The 10 mM stock solution of DHR was prepared in DMSO, aliquoted, and stored under nitrogen at −20 °C. The MnTnHex-2-PyP^5+^ (MnP) was synthetized as described previously [20] (charges are omitted for clarity throughout the manuscript).

### 2.2. Cell Culture

The human renal cancer cell line 786-O was obtained from ATCC (Manassas, VA, USA) and cultured in RPMI-1640 medium, supplemented with 10% fetal bovine serum (FBS) and 1% pen/strep. Cells were maintained at 37 °C, under a humidified air atmosphere containing 5% of CO_2_.

### 2.3. Crystal Violet (CV) Staining Assay

Cell viability was firstly evaluated by the CV staining assay. Cells were seeded in a 96 well plate and incubated for 24 h. Afterward, cells were incubated for 12, 16, 24, or 48 h with MnP (0.1–25 μM) in medium containing 2% FBS or 10% FBS. CV assay was carried out using 2% FBS in order to select a non-toxic concentration of MnP to be used in the subsequent experiments since low FBS medium is more appropriate for cell migration assays. The highest FBS concentration was chosen to better mimic the in vivo conditions. H_2_O_2_ (10 mM) was used as a positive control. The CV assay was then carried out according to previously described protocols [21,22]. Absorbance values for untreated control cells correspond to 100% cell viability. For this assay, two to three independent experiments were carried out. Three to six replicate cultures were used in each independent experiment. 

### 2.4. MTS Reduction Assay

The MTS assay was carried out as a complementary assay to evaluate the cell viability of MnP-treated cells in medium containing 2% FBS. The MTS reduction assay was carried out according to a previous protocol [23]. Briefly, 786-O cells were seeded at a density of 8 × 10^3^ cells per well in 100 μL of culture medium in 96-well plates and incubated for 24 h. The culture medium was removed and cells were treated with MnP (0.25 and 5 μM) for 12 or 24 h in medium containing 2% FBS. Following the drug treatment, the medium was removed, each well was rinsed with PBS and cells were incubated for 2 h with 100 μL of culture medium and 20 μL of CellTiter 96^®^ AQueous One Solution Reagent. Absorbance was measured at 492 nm. H_2_O_2_ (10 mM) was used as a positive control. Absorbance values for untreated control cells correspond to 100% cell viability. Two independent experiments were performed each one comprising six replicate cultures. 

### 2.5. Cell DNA Content Analysis

The cell cycle distribution of cells treated with MnP was determined by flow cytometry. This assay was carried out according to Guerreiro et al. [24] and Silva et al. [25]. Briefly, 1.5 × 10^5^ cells were cultured in 6-well plates for 24 h in 10% FBS medium. Cells were exposed to MnP (0.25 and 5 µM) in medium containing 2% FBS for 24 h. Cells were harvested using 5 mM EDTA in PBS, washed with PBS and fixed in 80% ethanol. After RNase A-treatment (20 µg/mL) and PI (10 µg/mL) staining, cells were analyzed using a FACSCalibur flow cytometer (Becton Dickinson, San Jose, CA, USA). Data acquisition and analysis were performed using CellQuest software (Becton Dickinson, San Jose, CA, USA) and FlowJo (Tree Star, San Carlos, CA, USA), respectively. Three independent experiments were performed.

### 2.6. Apoptosis Assay

The percentage of apoptotic cells was assessed using the dead cell apoptosis kit with Alexa^®^ Fluor 488 Annexin V and PI for flow cytometry (Molecular Probes, Eugene, OR, USA), according to the manufacturers’ instruction. Approximately 1.5 × 10^5^ cells were cultured in 6-well plates for 24 h. Afterward, the cell culture medium was replaced by a medium with 2% FBS, cells were exposed to MnP (0.25 and 5 µM) for 24 h, harvested by soft trypsinization (0.5 mg/mL) and washed twice with cold PBS. Cells were analyzed by flow cytometry (FACSCalibur flow cytometer - Becton Dickinson, San Jose, CA, USA). Data acquisition and analysis were performed using CellQuest software (Becton Dickinson, San Jose, CA, USA) and FlowJo (Tree Star, San Carlos, CA, USA), respectively. Three independent experiments were performed.

### 2.7. Intracellular ROS Evaluation

Intracellular ROS analysis was performed using the DHR probe. Approximately 2 × 10^5^ cells were cultured in 6-well plates in a complete culture medium. After 24 h, the medium was replaced, and 786-O cells were exposed to MnP (0.25 and 5 µM) for 12 h in medium containing 2% FBS. The medium was removed, cells were rinsed with warm PBS, detached with TrypLE™ Express Enzyme solution and incubated with DHR (10 μM) in FBS-free media for 30 min at 37 °C and 5% CO_2_. Cells were analyzed using a FACSCalibur flow cytometer (Becton Dickinson, San Jose, CA, USA). Data acquisition and analysis were performed using CellQuest software (Becton Dickinson, San Jose, CA, USA) and FlowJo (Tree Star, San Carlos, CA, USA), respectively. The median of DHR fluorescence intensity of approximately 2 × 10^4^ cells per condition was used to compare the intracellular ROS levels. H_2_O_2_ (10 mM) was used as a positive control. Four independent experiments were performed.

### 2.8. Cytokinesis-block Micronucleus (CBMN) Assay

Approximately 2 × 10^3^ cells were cultured in 8-well Lab-Tek™ II Chamber Slide™ System (Nunc) for 24 h. Afterward, the cells were incubated with MnP (0.25 and 5 μM) for 24 h. Mitomycin C (0.75 μM) was used as a positive control. The cells were then washed with culture medium and incubated with cytochalasin B (4.5 µg/mL) for 26 h. Afterward, the cells were rinsed with PBS and the slides were fixed with ice-cold methanol for 20 min at −20 °C. After air drying, the slides were stained with Giemsa as described in [26] and coded for microscope analysis. Three independent experiments were carried out and two replicate cultures were used in each independent experiment. For the assessment of micronuclei (MN) frequency, 1000 binucleated (BN) cells with a well-preserved cytoplasm were scored using 1000× magnification on a light microscope (Olympus BX43F), according to described criteria [27]. The frequency of micronucleated binucleated cells (MNBN) and the total number of MN were used as genotoxicity indices. The decrease in cell proliferation was evaluated by two standard indices: the percentage of binucleated cells (% BN) and the nuclear division index (NDI, [27,28]). For these indices, 500 cells were classified according to the number of nuclei using a 500× magnification in a light microscope (Olympus BX43F).

### 2.9. In Vitro Wound-Healing Assay

The in vitro wound-healing assay was optimized according to Liang et al. [29]. Briefly, 2 × 10^5^ cells were seeded in 24-well plates and cultured in complete medium. After 24 h each well was scratched using a 200 µL pipette tip, leaving a gap of approximately 0.8 mm in width. Cells were then rinsed twice with PBS to remove the detached cells and cell debris and incubated with MnP (0.25 μM) in medium containing 2% FBS. Wound closure was evaluated with an Olympus CKX41 inverted microscope. Photographs of the same areas of the scratch were taken using a 4× objective with an Olympus SC20 camera at 0, 8 and 12 h. The scratches width was measured using ImageJ software (National Institutes of Health, Bethesda, MA, USA). Wound closure was calculated in relation to the initial distance between the two scratches edges which was considered as 0% of wound closure. Three independent experiments were performed.

### 2.10. Chemotaxis

The chemotactic migration of 786-O cells was evaluated in 24-well plates with transwell inserts with transparent PET membranes containing 8-µm pores (BD Falcon, Bedford, MA, USA). Cells (1 × 10^5^ cells in 2% FBS medium) were seeded on the top of the insert, and a complete medium was placed in the lower chamber of the culture well. The MnP (0.25 µM) was added to both chambers, and cells were incubated for 12 h. To measure random individual cell migration, a control experiment was performed using the same experimental conditions but adding 10% of FBS to both chambers. After the incubation period, non-migrating cells were gently removed from the upper compartment with a cotton swab. Cells present in the bottom of each membrane were fixed with cold 96% ethanol and stained with 0.1% crystal violet in 10% ethanol. The number of cells was counted in five separate fields by light microscopy using an Olympus SC20 microscope with a 10× objective. The results were expressed as percentages relative to non-treated control cultures. Three independent experiments were performed.

### 2.11. Statistical Analyses

Differences in mean values of the results were evaluated by the two-tailed Student’s t-test, after assessing normality and homogeneity of the variances. All analyses were performed with the SPSS statistical package (version 25, SPSS Inc. Chicago, IL).

## 3. Results

### 3.1. MnP Decreases 786-O Cell Viability

**Figure 1:**
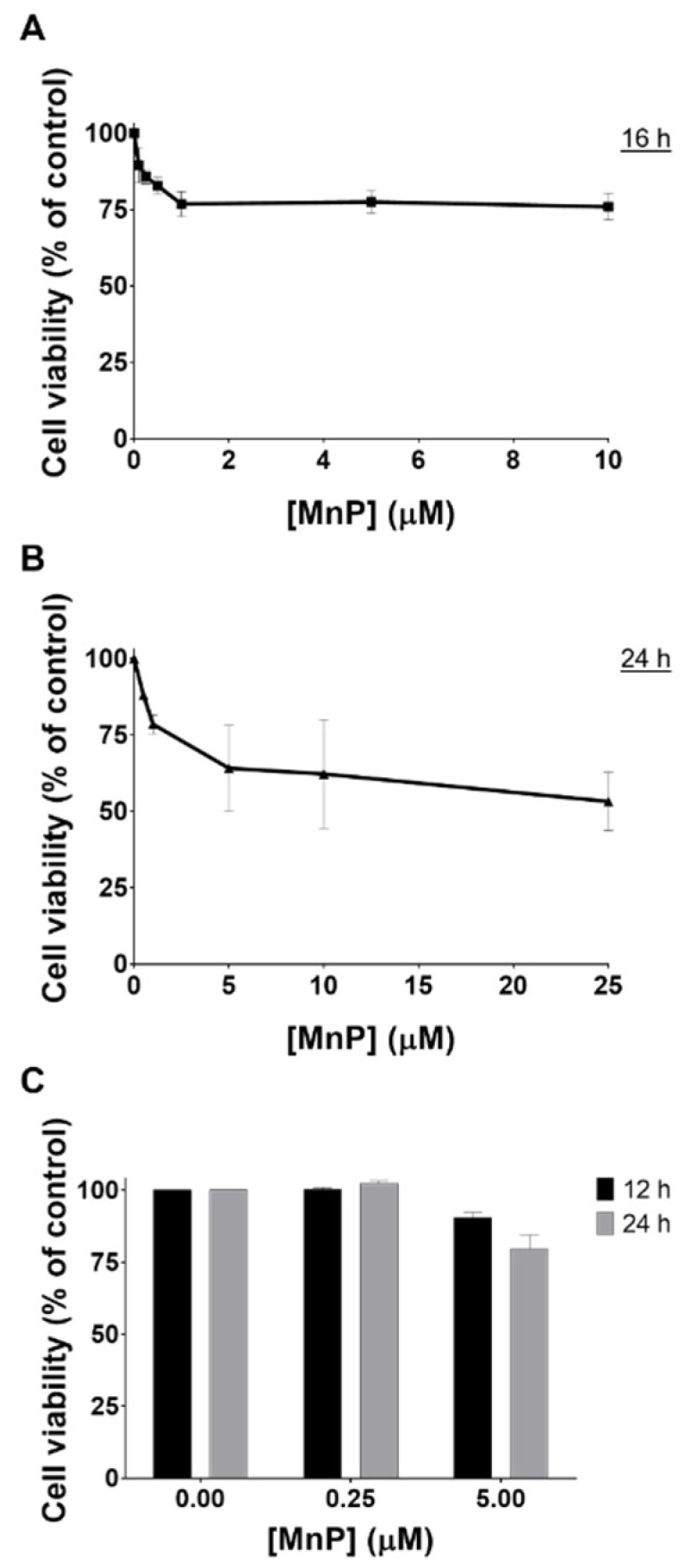
Cytotoxic effects of MnP (0.1–25 μM) in 786-O cells with 2% FBS. The viability of MnP-exposed cells (12–24 h) was evaluated by CV (**A**,**B**) and MTS (**C**) assays. Values represent mean ± SD (n = 2–3) and are expressed as percentages of the non-treated control cells.

**Figure 2:**
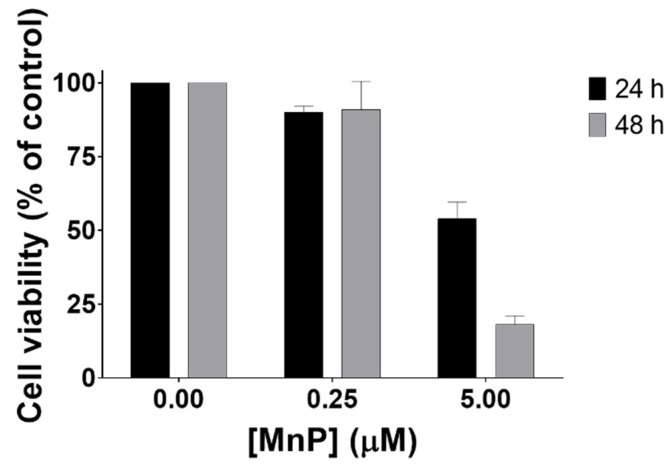
Cell viability of 786-O cells exposed to MnP (0.25 and 5 μM), using 10% FBS. The cell viability of MnP-exposed cells (24 and 48 h) was evaluated by CV assay. Values represent mean ± SD (n = 3) and are expressed as percentages of the non-treated control cells.

The effect of MnP treatment on cell viability was assessed through different methodologies. Exposure to MnP (0.1–25 μM) induced a decrease in cell viability as assessed by the crystal violet (CV) staining assay (Figure 1A,B) at different exposure times (16 and 24 h). The results obtained with the MTS reduction assay were similar (Figure 1C). The viability of cells exposed to 0.25 μM of MnP remained unchanged for both exposure times (12 and 24 h). However, when 786-O cells were exposed to a higher concentration of MnP (5 μM), a time-dependent cytotoxic effect was observed. The viability of MnP-exposed 786-O cells in medium containing 10% FBS was also assessed by CV assay at 24 and 48 h periods (Figure 2). For this purpose, two different concentrations of MnP (0.25 and 5 μM), below and above the threshold of cytotoxicity were selected based on the previous results obtained from the cell viability assay with 2% FBS. These results show a remarkable and statistically significant decrease in cell viability for the MnP concentration of 5 μM (*p* < 0.001 for both exposure times). 

The viability assays also allowed the selection of the MnP concentration of 0.25 μM for the cell migration studies, since no cytotoxic effects were found at this concentration level. As dying cells poorly migrate, the use of non-cytotoxic concentrations is a requisite when testing cell migration [30,31].

### 3.2. MnP Increases 786-O Cell Death

**Figure 3:**
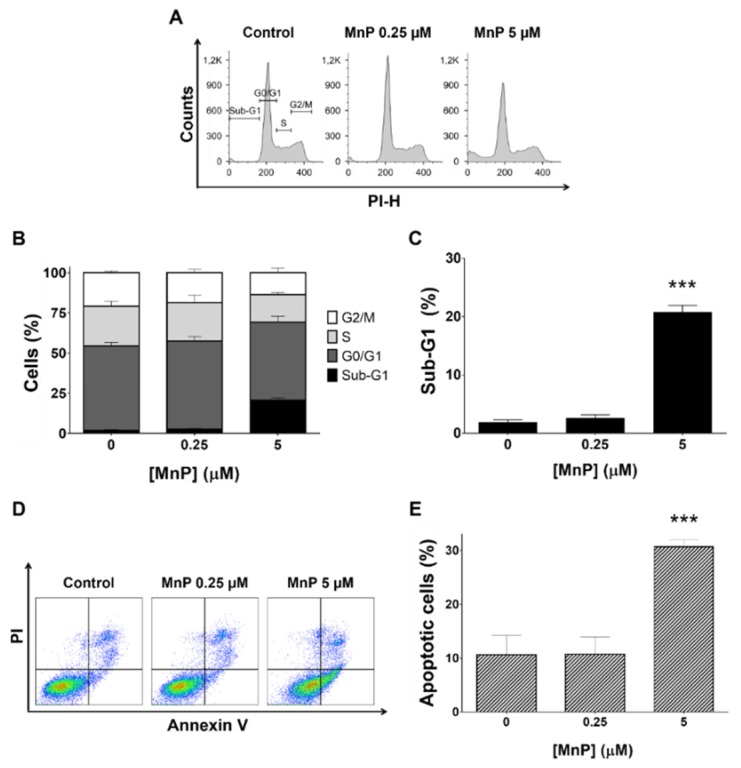
Effect of manganese porphyrin (MnP) on the cell cycle progression of 786-O cells. Cellular DNA content was analyzed by flow cytometry after 24 h incubation with MnP. (**A**) representative flow cytometry histograms. (**B**) sub-G1, G0/G1, S, and G2/M populations summary results. (**C**) sub-G1 population percentage. Percentage of apoptotic cells determined by PI and Annexin V staining (**D**,**E**) with representative flow cytometry dot-plots (**D**) and summary results show the percentage of apoptotic cells (**E**). Values represent mean ± SD (n = 3), *** *p* < 0.001 (Student’s t-test).

The impact of MnP in the cell cycle progression and cell death of 786-O cells was investigated by assessing the cellular DNA content using PI stain in fixed cells (Figure 3A). The exposure to MnP (5 μM, 24 h) led to a significant increase of 19% in the sub-G1 population when compared with the untreated cells and, with a consequent decrease in the S and G2/M populations (Figure 3B,C). The lower concentration of MnP (0.25 μM, 24 h) led to a cell cycle distribution similar to that of control cells (Figure 3A,B). All three independent experiments carried out led to coherent results.

The induction of apoptosis was determined by flow cytometry analysis of cells stained with Annexin V and PI. Representative graphs obtained by flow cytometric analysis of the cells are shown in Figure 3D. Exposure to MnP (5 μM, 24 h) showed an increase in apoptotic cells of ~20% (*p* < 0.001 vs non-treated control cells, Figure 3E) which is consistent with the observed increase of the sub-G1 population. The MnP (0.25 μM, 24 h) did not change the % of apoptotic cells compared with non-treated control cells. 

### 3.3. MnP Increases Intracellular Levels of ROS in 786-O Cells

**Figure 4:**
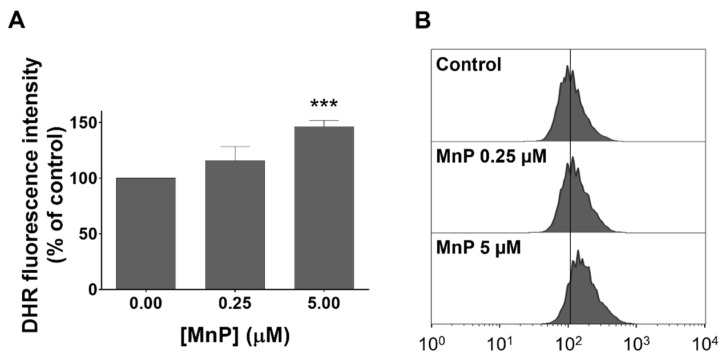
Exposure to MnP led to an increase in intracellular ROS in 786-O cells. The intracellular ROS levels after 12 h of exposure were detected by flow cytometry using the DHR probe. (**A**) Values represent mean of the median fluorescence intensity ± SD (n = 4), *** *p* < 0.001 (Student’s t-test). (**B**) Representative histograms from one assay are shown.

The level of intracellular ROS was analyzed by flow cytometry using the DHR fluorescence probe. A concentration-dependent ROS increase upon exposure to MnP when compared with non-treated control cells was observed (Figure 4). For the lowest concentration of MnP (0.25 µM) an increase in the individual cell fluorescence intensity of approximately 16% (*p* = 0.05) was detected. A considerably higher fluorescence increase of about 46.5% (*p* < 0.001) was observed for the highest concentration tested (5 µM). The four independent experiments performed led to coherent results.

### 3.4. MnP Does Not Induce Genotoxicity in 786-O Cells

**Figure 5:**
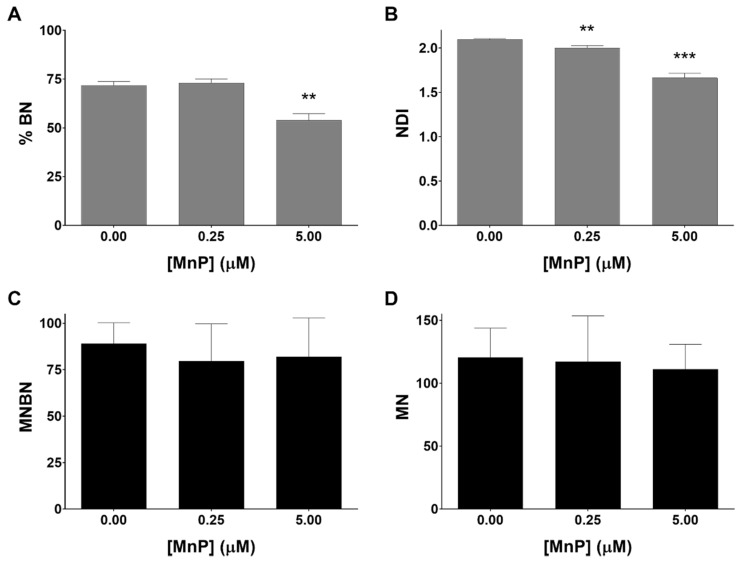
The effects of MnP on proliferative indices (**A**,**B**) and induction of micronuclei (**C**,**D**) in 786-O cells evaluated by the cytokinesis-block micronucleus (CBMN) assay. Percentage of binucleated cells (% BN) (**A**), nuclear division index (NDI) (**B**), frequency of micronucleated binucleated cells (MNBN) in 1000 BN cells (**C**) and a total number of micronuclei (MN) in 1000 BN cells (**D**) are shown. Values represent mean ± SD (n = 3), ** *p* < 0.01 and *** *p* < 0.001 (Student’s t-test).

A further aspect of the present work was to assess the genotoxic potential of the MnP treatment in 786-O cells. It is important to mention that many classical agents used in cancer treatment have a DNA damage-based mechanism of action, inflicting excessive DNA damage that is further converted into toxic lesions [32]. In this sense, the cytokinesis-block micronucleus assay was selected. The exposure to MnP at the highest concentration (5 μM) led to a reduction in the frequency of binucleated (BN) cells of approximately 18% (Figure 5A). Additionally, the exposure to MnP decreased the nuclear division index (NDI), which also reveals the effect of the MnP in the cell division of this cell line (Figure 5B). The lowest concentration of MnP tested (0.25 μM) did not show a relevant impact in terms of cell division. Regarding the formation of micronuclei, the total number of micronuclei (MN) in control cells (Figure 5D) presented a level similar to that of a previous study performed in 786-O cells using different methodologies [33]. Importantly, the frequency of micronucleated binucleated cells (MNBN) and the total number of MN cells did not show significant differences in the presence of MnP, when compared with non-exposed control cells (Figure 5C,D).

### 3.5. MnP Reduces the Chemotactic 786-O Cells’ Migration

**Figure 6:**
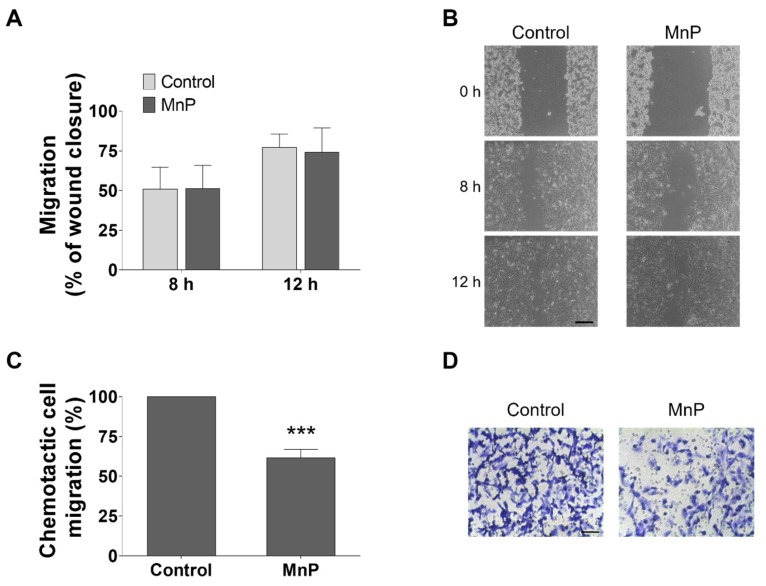
Effect of MnP treatment (0.25 µM) on 786-O cell motility. Collective cell migration was evaluated by the wound-healing assay (**A**,**B**) and chemotaxis was measured using a transwell assay (**C**,**D**, 12 h). Representative microscopy images (**B**,**D**). Scale bars 200 μm (**B**) and 80 μm (**D**). Values represent mean ± SD (n = 3), *** *p* < 0.001 (Student’s t-test).

Collective cell migration was assessed by the wound-healing assay. Treatment of 786-O cells with MnP (0.25 µM) did not alter collective cell motility (Figure 6A,B). However, different results were observed in chemotaxis assessed by the transwell assay using FBS as the chemoattractant (Figure 6C,D). The incubation with MnP (0.25 µM) significantly decreased the chemotactic migration to 61.5% ± 5.4 (*p* < 0.001), when compared with non-treated control cells. To confirm if migration through the transwell pores was indeed due to chemotaxis and not random motility, control experiments were carried out using the same % FBS in both the upper and lower compartments. In this case, no significant decrease was observed for cells exposed to MnP (0.25 µM) when compared with control cells (data not shown).

## 4. Discussion

Although many data have been published regarding the beneficial effects of MnPs in different pathologies, including cancer treatment, its influence in renal cancer has not previously been addressed. MnSOD and MnPs were shown to reduce the cell viability or induce cell death in different in vitro cancer models, including in breast cancer [34], skin cancer [35], prostate carcinoma [36] or colorectal cancer [37]. However, this cytotoxic effect in cancer cells is not always present as reported in other studies, including from our group [38], and depends on several contributing factors, including the type of porphyrin and its concentration as well as the cancer cell model chosen and respective cellular redox status. This work was carried out using 786-O cells, a well-established and characterized human cell line derived from primary clear-cell adenocarcinoma from a male patient. The ccRCC is the most common type of RCC and its incidence is higher in men than women. The selection of 786-O cells in this study was therefore based on the clinical and epidemiological data of RCC as well as in their specific cellular and genetic characteristics, including the inherent invasiveness and the defective *VHL* expression. Thus, 786-O cells are considered an appropriate model of RCC [39] and have been widely used in different types of research, such as proliferation [40], apoptosis [41], migration and invasion [42], metastasis [43] or therapy resistance [44] studies.

Previous pharmacokinetic studies of MnTnHex-2-PyP in in vivo models showed highest bioavailability in the liver, followed by the kidney. In mice, the administration of MnTnHex-2-PyP through different routes and at clinically relevant levels led to plasma and kidney concentrations in the micromolar range [45], which is in accordance with the concentrations used in our study. Moreover, this MnP is preferentially distributed to tumor relative to non-tumor tissues, and it accumulates predominantly in mitochondria, thus mimicking SOD2 (MnSOD) [18].

In the present work, a concentration- and time-dependent cytotoxic effect of the MnP in 786-O cells was clearly observed. The effect in cytotoxicity parallels a decrease in cell proliferation indices. Interestingly, 786-O cells were more sensitive to the toxicity of MnP when comparing to other cell lines previously studied, namely to Vero kidney cells [26], human breast cancer cells (MCF7 and MDA-MB-231, [38]), human inflammatory breast cancer cells SUM149 [46], or human epithelial colorectal adenocarcinoma cells Caco-2 [40]. Interestingly, different studies have pointed to higher cytotoxicity in cancer cells when compared with non-cancer models, both for MnTnHex-2-PyP and for other Mn(III) porphyrins [18,47].

Chemically-induced cytotoxicity in cancer cells is often accompanied by cell cycle arrest and apoptosis induction. Therefore, we aimed to explore the involvement of these mechanisms on the MnP toxicity. In this study, while MnP at 0.25 µM did not impact the cell cycle distribution, the highest concentration increased the sub-G1 population, which is usually related to DNA fragmentation and cell death. Such alteration was accompanied by an increase in apoptosis, which is also in accordance with the cell viability results previously discussed.

In order to characterize the influence of MnP on the redox balance of 786-O cells, the DHR fluorescence probe was used. In the present study, a concentration-dependent increase in intracellular ROS levels was observed. While disproportionating superoxide anion, SODm generates H_2_O_2_ and it is known that in peroxidase-containing cells, H_2_O_2_ oxidizes DHR [48,49]. In addition, H_2_O_2_ can also be produced by redox cycling of MnP with intracellular reducing agents, such as ascorbate, glutathione or cysteine [18]. Moreover, low activity of enzymes capable to detoxify H_2_O_2_, such as glutathione peroxidase [50], catalase [11], or both [12], was reported in RCC. This evidence, along with the insignificant catalase-like activity of MnPs [51], can justify the ROS increase observed with this fluorescence probe.

The MnPs have shown to be associated with a low in vitro toxicity potential demonstrated in studies performed with non-tumor cell lines as well as in in vivo studies [18,52]. This important feature has already been reported for MnP (up to 25 µM) in Vero cells, a non-human primate renal cell model [26]. These differential effects could be attributed to the higher level of intracellular ROS, particularly H_2_O_2_ in cancer cells when compared with non-tumor cells. In addition, the cellular antioxidant defenses in cancer cells are generally lower than in normal tissues [18]. Thus, the increase in intracellular levels of H_2_O_2_ induced by the inherent redox mechanism of MnPs, along with the limited ability of cells to detoxify this species, could lead to cell death. Our data suggest that when 786-O cells were treated with the lowest concentration of MnP, the increase in H_2_O_2_ was not high enough to trigger cell death mechanisms. On the other hand, with the concentration of 5 µM of MnP the threshold of toxicity may be reached, and this mechanism may contribute to the differential cytotoxic effects observed.

To further explore the mechanisms of MnP toxicity, the genotoxicity was also assessed. There are only a few published reports regarding the genotoxicity of MnPs, and those reports aimed to evaluate their safety profile. Recently, Gad S. C. et al. performed two nonclinical safety studies with other MnPs, MnTE-2-PyP [53] and MnTnBuOE-2-PyP [54] that included different genotoxicity assays, and concluded that in both studies, MnPs are negative for genotoxicity. Herein, the genotoxicity of MnP was assessed through the CBMN assay. This MnP, at the highest concentration (5 µM), impacted the normal cell division of 786-O cells, which is in accordance with the results of the cytotoxicity assays. No genotoxicity was observed with the MnP, which is in accordance with the results reported in the literature for other MnPs using different cell models and experimental conditions [54]. Therefore, the toxic effects herein observed for the highest concentration of MnP do not seem to involve genotoxicity, either directly or indirectly, through the increase of ROS intracellular level.

RCC is associated with a high potential of metastases [55]. The formation of metastases involves several biological mechanisms, including an increase in cell motility, which can be assessed by in vitro migration assays. Mechanistically, ROS may participate in various signaling pathways associated with cell migration [56,57]. In this work, cell migration was evaluated by two distinct methodologies. The wound-healing assay provides insights into collective cell motility, evaluating the movement of cells across a horizontal surface with the preservation of functional cell–cell junctions [30,58]. Conversely, the chemotaxis assay evaluates the migration of individual cells through a membrane pore toward a chemoattractant [58]. In our study, the MnP did not lead to a reduction in collective cell motility, but it significantly decreased the chemotactic migration. The MnP could act either as a direct migration modulator or indirectly through ROS, which can modulate several migration signaling pathways. The effect of the MnP in cell migration could be also related to the modulation of the nuclear transcription factor kappa B (NF-κB), hypoxia-inducible factors (HIF) and mTOR pathways, which have been reported to influence cell migration [59,60,61].

## 5. Conclusions

We demonstrated that MnP exhibits differential concentration responses in 786-O cells. At 0.25 µM, this compound did not display any cytotoxicity. Interestingly, however, it has the ability to reduce individual cell migration. Furthermore, at 5 µM, MnP is clearly cytotoxic, increases the % of apoptosis and decreases cell division/proliferation indices. Importantly, it also enhances the level of intracellular ROS of 786-O cells which can be a possible mechanism of MnP action. Taken together, these observations suggest that MnP may have a beneficial impact on reducing the viability and migration of renal cancer cells. This in turn, warrants further studies, namely with other invasive renal cancer cell lines as well as with in vivo rodent experiments to support the efficacy of SODm in general, and of MnP in particular, in the context of human renal cancer.
